# XXIX Sespo congress, Santiago de Compostela, Spain, October 25-26, 2024

**DOI:** 10.4317/jced.1122335667804

**Published:** 2025-06-01

## ABSTRACTS OF THE CONGRESS

### **CONFERENCE 1:** Workshop on the clinical diagnosis of caries using the ICDAS system

**Carmen Llena Puy**

ICDAS II (International Caries Detection and Assessment System) is an international consensus-based caries detection and diagnosis system for clinical practice, research and public health. The system aim is to develop a visual method for the detection of caries in its earliest stages and to detect the severity and level of caries activity. The system comprises two digits, the first from 0 to 8 corresponding to the restoration and sealant code, the number 9 to the missing tooth code. The second digit corresponds to the clinical diagnosis of caries and comprises seven categories; code 0 corresponds to a healthy tooth, codes 1 and 2 for caries limited to enamel, code 3 for cavitated caries in enamel, code 4 corresponds to a dark shadow visible through the enamel, with or without microcavity and codes 5 and 6 correspond to caries with exposed dentine. This caries lesion classification system allows for the use of the ICCMS (International Caries Classification and Management System) which aims to provide a standardized method for integrating and synthesizing tooth and patient information, including caries risk status, in order to plan, manage and monitor caries in clinical practice. Full information on the ICCMS and its implementation can be found at the following link. https://www.iccmsweb. com/ The workshop will review the standardized ICDAS codes and train the clinical diagnosis of lesions with different extent and activity.

### **CONFERENCE 2:** Health and physical activity: How can we enhance their potential?

**Javier Rico Díaz**

There is abundant scientific evidence on the positive effects of physical activity on health. The World Health Organization insists on the importance of acquiring and maintaining an active lifestyle that, if possible, incorporates daily physical activities combining aerobic and strength work, with a minimum of 300 minutes of weekly practice in schoolchildren and 150 minutes in adults. Despite the fact that the population is aware of these benefits, the 5th Eurobarometer of Sport and Physical Activity (2022) shows that 31% of the population of the European Union and 45% of the Spanish population is physically inactive, with the consequences that this has both for health and for the public coffers. Could it be an object of reflection to establish in the school stages some standards of motor development and physical condition, that have an impact on a greater and better physical-sports practice in the school context, or would it be too interventionist? Could this lay the foundations for an active life throughout the life cycle? What could be contributed from the health, educational, business or community spheres, among others? What seems to be clear is that any intervention that aims to strengthen the binomial health and physical activity requires action from the earliest ages, taking advantage of all available resources, incorporating new ones and establishing a common, coordinated and clear thread for both institutions and the business and associative fabric, as well as for citizens in general.

### **CONFERENCE 3:** The key points of an epidemiological study of oral health

**José María Montiel Company**

Epidemiological studies of oral health are a key and ideal tool to obtain a clear view of the prevalence of diseases that affect the oral cavity. Like any observational and cross-sectional study, guaranteeing the representativeness of the sample and the sample size are essential points to ensure the validity of the study. In this presentation we will also address the importance of calibrating the explorers who participate in a study and ensuring validity and reliability in the measurements. With all these guidelines, a basic methodology divided into phases to be followed to successfully carry out an epidemiological study will be proposed.

### **CONFERENCE 4:** Teleodontology

**Antonio Expósito Delgado**

Teleodontology is a branch of dentistry that uses information and communication technologies to provide dental services at a distance. This modality has gained popularity, especially during the COVID-19 pandemic, due to the need for social distancing and the limitation of face-to-face consultations. Current applications of teleodontology are diverse and span several areas of dental care. Among the main applications are: • Virtual consultations: these allow patients to receive initial advice and diagnosis without the need to travel to an office. This is especially useful for people in rural areas or those with reduced mobility. • Diagnosis and follow-up: Dentists can evaluate images and videos submitted by patients to diagnose dental problems and follow up on ongoing treatments. • Education and training: Teleodontology facilitates continuing education for dental health professionals and training for patients on preventive care. • Simple treatments: In some cases, basic treatments can be performed remotely, such as guidance for handling dental emergencies. • Electronic Health Record (EHR): The management of patient information is optimized through the use of EHRs, allowing fast and secure access to clinical data.

### **CONFERENCE 5:**How can we decipher food labels?

**Maruxa Zapata Cachafeiro**

Nowadays, the vast majority of the food we can find in a supermarket is packaged. Shopping is becoming an increasingly difficult activity, especially if we want it to be healthy. The food industry offers us an infinite number of products, often very similar to each other, and made up of huge lists of ingredients. Although nutrition labelling is regulated, it can be difficult to understand. This conference will analyse what information must and can be included on a food label according to the regulations, and will explain the different ways that the food industry uses so that we often form a better image of their product than what it really is.

### **CONFERENCE 6:**Materials used in Minimal Intervention Dentistry

**Yolanda Martínez Beneyto, Teresa Almerich Torres**

Minimal Interventional Dentistry (IMD) focuses on preserving as much healthy tooth structure as possible, using less invasive approaches. Advances in materials have been instrumental in supporting this philosophy, allowing for more conservative and effective treatments. Among the most important materials are glass ionomers, which release fluoride and have adhesive and remineralising properties, promoting long-term dental health. These materials are ideal for low-risk caries restorations, especially in paediatric patients or those with special needs. Silver diamine fluoride (SDF) is of great importance in minimally invasive dentistry due to its ability to stop caries progression in a non-invasive manner. This material works by inhibiting bacterial growth and remineralising the affected tooth structure, without the need to remove decayed tissue, making it an ideal option for patients at high risk of caries or those who cannot undergo invasive procedures. In addition, it is simple and quick to apply, making it suitable for use in community and paediatric dentistry. The SDF reinforces the preventive and conservative approach of minimal intervention dentistry, offering an effective and less traumatic solution for caries control. Finally, biomaterials such as hydroxyapatite and mineral trioxide add bioactive properties, promoting tissue regeneration and further integration with tooth structure. These materials allow OMI professionals to offer treatments that respect the biology of the tooth and minimise invasive intervention, aligning with the preventive and restorative objectives of this discipline.

### **CONFERENCE 7:**The contribution of dentistry to the problem of antibiotic resistance

**Olalla Vázquez Cancela**

Antibiotic resistance is increasing worldwide, to the point where it is now one of the main threats to Public Health. In the fight against the advance of this threat, reducing consumption is a crucial element. The factors that influence the prescription, over-the-counter dispensing and consumption of antibiotics have been under study for years. Most of these factors are potentially modifiable (knowledge, attitudes, professional-patient relationship) through specific interventions. Dentists prescribe around 10% of all antibiotics consumed and it is estimated that more than 70% of these prescriptions, according to current clinical guidelines, would not be considered appropriate. As with other groups such as doctors or pharmacists, identifying the factors associated with inappropriate prescribing would enable the development of specific strategies aimed at optimising the use of antibiotics. To identify dentists' knowledge, attitudes and perceptions regarding the use of antibiotics in dentistry and their relationship with inappropriate prescribing. A systematic review was carried out following the PRISMA criteria. Focus groups were held until information saturation was reached. The magnitude of inappropriate antibiotic prescribing by dentists and the influence of knowledge, attitudes and perceptions on the quality of prescribing were quantified through a crosssectional questionnaire study following the STROBE guidelines. The factors that influence the prescription of antibiotics by dentists are modifiable. The systematic review, which included 37 articles, identified attitudes such as complacency, lack of confidence, the need to postpone the dental procedure and fear as the main factors influencing the prescription of antibiotics by dentists. Through the 7 focus groups carried out, in addition to the aforementioned factors, symptoms of dentist burnout or a history of biological treatment in the patient were identified as determinants of prescription. Furthermore, in the cross-sectional study, in which 878 dentists participated, it was evident that half of the participants had inappropriate antibiotic prescribing habits in more than 28.6% (10/14) of the clinical situations presented (interquartile range 57–79%). The quality of prescribing improved when resistance was perceived as a public health problem (OR 0.88, 95% CI: 0.79–0.97), and decreased in response to fear (OR 1.12, 95% CI: 1.07–1.18) or the pursuit of financial gain (OR 1.07, 95% CI: 1 .01–1.14). The prescription of antibiotics by dentists is predominantly influenced by modifiable factors. These findings highlight the importance of educational interventions that emphasise the fundamental role of dentists in the responsible use of antimicrobials. Just as dentists are leaders in the promotion of oral health, they can also play a crucial role in promoting the appropriate use of antibiotics, becoming key agents in the fight against antimicrobial resistance.

### **CONFERENCE 8:**Strategies for smoking cessation in the dental surgery

**Agustín Montes Martínez**

Tobacco consumption is the leading cause of preventable mortality worldwide. The WHO estimates that in the 21st century, it could kill up to 1 billion people around the globe. In Spain, it is estimated that 56,000 people die each year from diseases primarily affecting the circulatory system, cardiovascular system, and cancer. However, it also causes damage to oral health. There is no other preventable cause of disease with such a significant impact on the population's health as this, which makes it imperative to take action if we want to improve public health. The International Dental Federation highlights the important role that dentists play in this process: "As oral health professionals, dentists have various roles to play in global efforts to control tobacco use, including serving as role models, acting as doctors, educators, scientists, leaders, opinion and alliance makers." There are multiple strategies to help smokers quit tobacco. One of the most common in primary healthcare, but also used in specialized care, is the 5A strategy: Ask, Advise, Assess, Assist, and Arrange. To achieve the ultimate goal, different complementary approaches are used: Cognitive, Behavioural, and Pharmacological. In cases where a smoker is not willing to quit, their motivation can be increased using the 5R strategy: Relevance, Risks, Rewards, Roadblocks, and Repetition.

### **ORAL COMMUNICATION 1:**Evaluation of the knowledge of early childhood and primary education teachers about dental trauma: a cross-sectional study

**Nerea Rodríguez Pareces, Almudena Rodríguez, Paula Fernández, Marta Mulero, Maruxa Zapata Cachafeiro**

 Introduction:

 The high prevalence of traumatic dental injuries (TDIs), especially in school-aged children, as well as the importance of a quick and adequate management of them to increase the prognosis of the tooth, justify the need for teachers to know some basic concepts to be able to provide emergency management of TDIs in an adequate way.

Objectives:

 The aim of this study is to assess the level of knowledge about dental trauma among pre-school and primary school teachers.

Material and Methods:

 A cross-sectional observational study was carried out. An online, self-completed questionnaire was used, addressed to pre-school and primary school teachers. Sociodemographic and professional variables were collected, as well as knowledge and attitudes related to their performance with TDIs. The data obtained were analysed using descriptive statistical analysis, Fischer’s and χ2 test. A dependent variable was created from the responses to the practical cases to assess the quality of performance.

Results:

 The study sample consisted of 370 teachers. 53,6% of the teachers had an average immediate performance quality of the TDIs. There were no significant differences in performance quality among teachers according to gender, having children, years of professional experience, previous experience of TDIs, teaching speciality and physical education. Teachers who had received previous TDIs training showed better performance quality (p=0,005).

Conclusions:

 Our results reveal that teachers’ knowledge of TDIs is not adequate, and educational interventions aimed at improving teacher’s knowledge of immediate care of TDs would be necessary.

### **ORAL COMMUNICATION 2:**Look at that mouth-study and community intervention in oral hygiene in children and adolescents of Ourense

**María Asunción Pérez López, María Jesús Pereira Gil, Carlos Garrote Vázquez, Lorena Quintas Fírvida, María Sol Mouriño Vázquez, Miguel Franco Pérez**

 Introduction:

 Good oral health habits are most effective when established in youth, as they tend to last a lifetime. In Galicia, the latest studies date back to 2010 and lacked focus on preventive habits.

Objectives:

 Conduct community intervention and research to measure children’s and adolescents’ knowledge and habits regarding oral hygiene while also educating them.

Material and Methods:

 Educational talks were given to 63 Ourense schools to 2000 students from 4º EP (1327 students) and 2º ESO (673 students) during the 2023/2024 school year. Surveys before and after the sessions to assess their knowledge and habits; elaborated according to previous literature and validated by the Comité de Ética de Investigación de Galicia (CEImG). Descriptive analysis, visualization and differences contrasts of the results are performed.

Results:

 We collected reliable data on brushing, dental visits, diet, and the effects of piercings and drugs. Misconceptions about diet and piercings were corrected. The "habit-knowledge gap" is larger in adolescents than children; they know what to do but admit not doing it. Women show better habits.

Conclusions:

 Significant knowledge improvement post-intervention, proving the program’s effectiveness. Valuable data for academic research was collected. Identified "infantilization of oral hygiene”. The research-intervention approach balances theoretical work with practical health improvements.

### **ORAL COMMUNICATION 3:**Interventions to improve the use of antibiotics in dentists: systematic review and meta-analysis

**Julieta Méndez, Almudena Rodríguez Fernández, Marta Ferreira, Ulises Villasanti, Gloria Aguilar, Carlos Rios, Adolfo Figueiras**

 Introduction:

 In the broader context of the rational use of antibiotics, dentists are key players in the efforts to curb antimicrobial resistance, as they are estimated to prescribe 10% of the antibiotics consumed and inappropriate prescriptions could be between 50 and 80%, which can lead to an unnecessary increase in resistance.

Objectives:

 Our objective was to update the previous systematic review carried out by Löffler to include new interventions carried out in dentists and to study the effectiveness according to the type of intervention by implementing a meta-analysis.

Material and Methods:

 We conducted an electronic search in the databases: MEDLINE, SCOPUS, EMBASE, COCHRANE CENTRAL, LILACS, BBO up to 2023 to evaluate the effect of interventions to improve antibiotic prescribing in dentists. A systematic data synthesis and meta-analysis were performed. The PRISMA tool was used to prepare the report. The protocol was registered in PROSPERO CRD42023474664. The Evidence Project risk of bias tool was used to assess study quality. Study heterogeneity and publication bias were evaluated.

Results:

 The search initially found 1914 studies, which after the elimination of duplicates 1380 studies were reviewed by 2 independent reviewers, including a third reviewer to resolve differences. We included 23 studies on interventions to reduce antibiotic prescribing in dentists. The studies were published between 1997 and 2023, mainly in the UK. Of the 23 studies, 3 were trials and 20 were pre-post studies. Interventions in general in dentists are able to reduce inappropriate prescribing by 70% (95% CI: 33.3% to 86.4%), which is very significant. In the pre-post studies, it was 71% (95% CI 28.8% - 88.1%) I2 99.2%. In the RCT studies it was 63.9% (95% CI 41% - 78.1%) I2 0%. However, the greatest effect size was in audit-based interventions with Audit and Education at 73.3% (95% CI 44% - 87.4%) and Audit and Feedback at 75% (95% CI 33% - 91.4%) respectively. To the best of our knowledge, this is the first meta-analysis to evaluate the impact of different interventions to improve the quality of antibiotic prescribing in dentists. However, these results should be taken with caution because most of the included studies have methodological weaknesses, mainly because most of the studies are pre-post studies without concurrent control groups.

Conclusions:

 Our results indicate that interventions ‘in general’ in dentists are very effective in reducing inappropriate prescribing. However, they should be taken with caution because most of the included studies have methodological weaknesses, mainly because most of the studies are pre-post studies without concurrent control groups.

Clinical significance:

 Given the magnitude of the effect found, it is demonstrated that dentists are receptive to improving their prescription and, in addition to that, there is ample room for improvement.

### **ORAL COMMUNICATION 4:**Tooth loss, chronic diseases and health risk behaviours in Spanish adults: Results from the European Health Survey in Spain

**Francisco Negreira Martínez**

 Introduction:

 Tooth loss is an oral disorder that directly affects basic functions such as chewing, worsening oral health-related quality of life. According to the Global Burden of Diseases 2019, the estimated global prevalence of total tooth loss is nearly 7.0%.

Objectives:

 To evaluate the association between noncommunicable diseases, common risk factors, and tooth loss among Spanish adults.

Material and Methods:

 Data provided by the European Health Survey in Spain 2020 (EESE), representative at the national level, were used. Prevalence Odds Ratio (POR) and its 95% confidence interval were calculated.

Results:

 Significant associations were observed between tooth loss and all risk factors evaluated, except for ultra-processed food consumption, with PORs ranging from 1.12 for sedentary lifestyle to 2.28 for infrequent dental visits. Multiple chronic diseases such as diabetes, cardiovascular and liver diseases showed a significant increase in the risk of tooth loss.

Conclusions:

 Our results suggest that risk factors and chronic diseases such as smoking, alcohol consumption, sedentary lifestyle, liver and cardiovascular diseases, and diabetes are related to tooth loss. These findings highlight the importance of integrating oral health into health promotion policies. In terms of clinical relevance, it emphasizes the need for an anamnesis that identifies all health risk behaviours, including those not directly related to oral health, and their chronic conditions.

### **ORAL COMMUNICATION 5:**Oral microbiome and machine learning: an innovative approach for the precision diagnosis of periodontitis

**Berta Suárez Rodríguez, Alba Regueira Iglesias, Alba Sánchez Barco, Iryna Kuz, Triana Blanco Pintos, Inmaculada Tomás Carmona**

 Introduction:

 Machine learning algorithms can be applied to microbiome data for precision phenotyping. There is no research to date applying these artificial intelligence techniques in various oral niches to diagnose periodontitis.

Objectives:

 To evaluate the capacity of the supragingival, subgingival, and salivary microbiomes to diagnose periodontitis using random forest and applying a compositional data analysis approach.

Material and Methods:

 Searches were performed to identify Illumina V3-V4 sequencing studies on supragingival plaque, subgingival plaque, and/or saliva and periodontal health and/or periodontitis. Our analysis included 33 bioprojects. Sequences were processed with mothur, and taxonomy was assigned using an oral-specific database. Statistical analysis was performed with R-Bioconductor.

Results:

 The supragingival model (samples in health=200; periodontitis=457) comprised four amplicon sequence variants (ASVs; 4.44% of the pre-selected variables). After data augmentation and validation (health=67; periodontitis=153), the model showed an accuracy of 86.28%, sensitivity of 83.66%, and specificity of 88.57%. The area under the curve (AUC) was 0.88. In contrast, the subgingival model (health=151; periodontitis=714) required more ASVs (nine; 15.00%) and obtained lower performance parameters after validation (health=51; periodontitis=238; accuracy= 78.67%; sensitivity=76.89%; specificity=80.22%; AUC=0.84). Finally, the salivary model (health=468; periodontitis=313) consisted of four ASVs (8.00%) and its post-validation performance was similar to that of the supragingival plaque model (health=156; periodontitis= 105; accuracy=86.21%; sensitivity=84.76%; specificity= 87.18%; AUC=0.91).

Conclusions:

 Artificial intelligence machine learning models for supragingival, subgingival and salivary microbiomes discriminate with high accuracy the health of periodontitis, with supragingival and salivary media proving to be the best diagnostic tools.

### **ORAL COMMUNICATION 6:**Microbiome diversity in periodontal health: a comparison of supragingival plaque, subgingival plaque and saliva

**Alba Sánchez Barco, Alba Regueira Iglesias, Berta Suárez Rodríguez, Rosalía Fontenlos Mato, Noelia Seijas Otero, Inmaculada Tomás Carmona**

 Introduction:

 The studies on the periodontally healthy microbiome in the supragingival and subgingival bacterial plaques and saliva have methodological shortcomings.

Objectives:

 To analyse the supragingival, subgingival, and salivary microbiomes in periodontal health at the amplicon sequence variant (ASV) level in terms of diversity, applying a compositional approach and removing batch effects.

Material and Methods:

 Searches were conducted to identify Illumina V3V4 sequencing studies on supragingival plaque, subgingival plaque, or saliva in periodontal health. Metadata and sequences that met predefined criteria were included (22 bioprojects, 848 samples). Sequences were processed with mothur, and taxonomy was assigned using an oral-specific database. Statistical analysis was performed with R-Bioconductor.

Results:

 Supragingival plaque was richer than subgingival plaque and saliva (observed ASVs=612.00 vs. 479.00 and 445.00; 95% coverage=224.00 vs. 171.00 and 172.00; p<0.05). Subgingival plaque showed a more even distribution (Pielou=0.65 vs. 0.62; p<0.01) and saliva had a greater diversity and evenness (Shannon= 4.11 vs. 4.07; Pielou=0.65 vs. 0.62; p<0.05) than supragingival plaque. In multivariate analysis, samples were grouped by niche (p<0.0001). Finally, 121 ASVs (39.93% in the comparative) presented differential abundance between bacterial plaques, 212 (68.83%) among supragingival plaque and saliva, and 160 (51.61%) between subgingival and saliva.

Conclusions:

 In periodontal health, supragingival plaque is richer than subgingival and saliva; but the subgingival plaque is more even, and saliva is more diverse and even than the supragingival plaque. The microbial community structure differs between niches. In terms of differential abundance, the bacterial plaques are more similar to each other than to saliva.

### **ORAL COMMUNICATION 7:**Prevalence of tooth decay in adults with Down syndrome: an unprecedented finding

**Hoda Tayebi Hillali, Carolina Alejandra Muñoz Navarro, Lucía López Sande, Clara Rodríguez Díaz, Iván Varela Aneiros, Eliane García Mato**

 Introduction:

 Numerous studies have confirmed that the prevalence of caries in individuals with Down syndrome (DS) is lower than in the general population, although this finding has not been critically analysed based on the age of the evaluated individuals.

Objectives:

 To assess the prevalence of caries in a cohort of individuals with DS and analyse its variability according to age.

Material and Methods:

 A convenience study group was formed with 200 individuals with DS recruited from users of educational therapy centres in the Autonomous Community of Galicia. A control group of 200 non-syndromic individuals was established, matched by age range. In both groups, the number of active caries lesions (ICDAS >2) and fillings was recorded, and the cod-CO index was calculated.

Results:

 In children with DS (n=55), 11 caries were recorded compared to 34 in the control group. In adults with DS (n=145), the number of caries was 70, compared to 68 in the control group. The CO index in individuals with DS was 1.03, compared to 1.22 in the control group. In the DS group, the CO index increased progressively from 0.15 in the 0–5-year age range to 2.24 in those over 35 years, while in the control group, the maximum CO was 1.21.

Conclusions:

 Assuming the limitations derived from the sample size, children with DS have fewer caries than non-syndromic controls, while their prevalence in adults with DS is higher than in the general population.

### **ORAL COMMUNICATION 8:**Association of DNA methylation with oral cancer risk

**Cristina Isabel Sevilla García, Ana Rodríguez Ces, Ángel Salgado Barreira, María Piñeiro Lamas, Mercedes Suárez Cunqueiro**

 Introduction:

 DNA promoter methylation is one of the main epigenetic mechanisms involved in carcinogenesis1,2.

Objectives:

 To evaluate the overall and specific impact of DNA promoter methylation on the risk of oral cancer development.

Material and Methods:

 PubMed, EMBASE, Web of Science, and the Cochrane Library were searched for eligible studies. The methodological quality of the studies was evaluated according to the Newcastle-Ottawa Scale (NOS). The pooled odds ratios (ORs) and 95% confidence intervals (CIs) were calculated with R software.

Results:

 A total of 41 studies including 4218 OC patients and 3478 non-cancer controls were englobed in the metanalysis. Overall, a significant association was found between DNA promoter methylation and oral cancer risk (OR = 4.81, 95% CI = 3.85—6.0; P < 0.01). In addition, the pooled ORs showed a significant association between specific tumour-related genes and oral cancer risk: p16 (5.77; 95% CI = 3.95–8.45), ECAD (4.47; 9 5% C I = 2 .77–7.21), M GMT ( 3.85; 9 5%CI = 2.48–5.97), hMLH1 (OR=10.48; 95%CI = 1.04–106.1), p14 (3.22; 95%CI = 1.79–5.79), and p15 (4.73; 95% CI = 2.61–8.56).

Conclusions:

 A significant overall association was found between gene promoter methylation and oral cancer risk. Specifically, the promoter methylation of p16, ECAD, MGMT, p15, p14, and hMLH1 could display a significant role in oral carcinogenesis, acting as promising biomarkers for oral cancer prediction and prognosis.

### **ORAL COMMUNICATION 9:**Bayesian methods for estimating false responses in surveys: application to adolescent smokers in Ourense

**Miguel Franco Pérez, Miquel March Torras**

 Introduction:

 Surveys on sensitive topics, such as adolescent smoking, may generate false responses. Bayesian methods offer an alternative to address this issue and obtain credible estimates.

Objectives:

 1) Develop a Bayesian method to estimate dishonesty in responses and obtain credible re-estimations. 2) Assess if adolescents lie about their smoking habits. 3) Study geographical and gender differences in false response distribution. 4) Re-estimate the prevalence of smokers among adolescents in Ourense.

Material and Methods:

 Using Bayesian methods a hard priori was constructed based on the ETUDES survey and compared with data from the "¡Look at that mouth! 2024" project (n=673 2nd ESO students) through a three hidden variables model: true smokers, lying smokers, and non-smokers. Stratified by gender and municipality.

Results:

 Falsehoods were detected in the original data, generating credible re-estimations. Dishonesty was higher in large municipalities and among males. In smaller municipalities, original data proved more credible.

Conclusions:

 Bayesian methods provide a suitable framework for addressing dishonesty in adolescent smoking surveys, allowing for more accurate re-estimations. However, a solid priori survey is required to ensure the credibility of re-estimations.

### **ORAL COMMUNICATION 10:**Tolerance of a new multi-head sonic toothbrush in adults with autism spectrum disorder

**Carolina Alejandra Muñoz Navarro, María Pilar Domingo Remacho, Ana Lamas González, Raúl Crespo García, Eliane García Mato, Jacobo Limeres Posse**

 Introduction:

 Autism Spectrum Disorder (ASD) presents a complex context for effective toothbrushing. While some studies have analysed the efficacy of different toothbrushes in children with ASD, there are very few references concerning adults.

Objectives:

 To evaluate the tolerance of a new toothbrush in adults with ASD and the potential benefit of conducting desensitization sessions.

Material and Methods:

 The study was conducted at the ASPANAES centre in Santiago de Compostela, after obtaining the necessary informed consent and the Ethics Committee’s approval. A total of 28 adults with ASD participated, 61% of whom were male, with an age range of 17-55 years. For each participant, their diagnosis (DSM-V) and sensory profile (Brown and Dunn) were recorded. Using a multiheaded sonic toothbrush (Balene Duotech®), tolerance (Frankl scale) was determined for completing a 2-minute toothbrushing session. Users who did not complete the toothbrushing underwent up to 5 desensitization sessions with an occupational therapist, and tolerance was re-evaluated afterward.

Results:

 68% tolerated the toothbrush in the first session, despite all participants having moderate to high severity ASD, and 53% presenting a high sensory sensitivity pattern. After desensitization, the total acceptance rate was 89%.

Conclusions:

 The percentage of participants who tolerated the toothbrush was very high, regardless of the severity of ASD or sensory sensitivity patterns, further confirming the benefit of desensitization.

### **ORAL COMMUNICATION 11:**Impact of Electronic Cigarette Use on Gingivitis and Periodontitis: A Systematic Review with Meta-Analysis

**Daniela García Valdez, Mónica Pérez Ríos, Cristina Cándal Pedreira, Guadalupe García, Julia Rey Brandariz, Marta Mascareñas García**

 Introduction:

 In recent years, new forms of tobacco consumption have emerged, such as electronic cigarettes (e-cigs). Although their impact on oral health has been investigated, little has been studied about their relationship with gingivitis and periodontitis.

Objectives:

 The objective was to identify studies that assessed the relationship between e-cig consumption and the impact on the oral cavity, specifically gingivitis and periodontitis.

Material and Methods:

 A bibliographic search was conducted in biomedical databases using PRISMA Material and Methods. Observational studies that evaluated the effect of exclusive e-cig consumption on the diagnosis of gingivitis/periodontitis were included. Studies that determined the presence of gingivitis/periodontitis clinically through self-reporting were considered. Quality was analysed using the Newcastle-Ottawa scale. For studies with clinical diagnosis, a descriptive synthesis was performed, while the results of self-reported studies were meta-analysed as odds ratios (OR) using a random effects model. Statistical analysis was conducted with Stata v.17.

Results:

 A total of 636 studies were identified, with 19 included; 10 performed clinical assessments and 9 established diagnoses through self-reporting. E-cig consumers showed worse results in clinical parameters, and the analysis of self-reported studies identified that e-cig consumption increases the risk of gingivitis (OR: 1.71; 95% CI: 1.13-2.59), periodontitis (OR: 1.46; 95% CI: 1.17-1.83), and bone loss (OR: 1.44; 95% CI: 1.19- 1.75).

Conclusions:

 E-cig consumption appears to increase the risk of gingivitis and periodontitis. It´s recommended that professionals don´t suggest e-cigs as a method for tobacco cessation.

### **ORAL COMMUNICATION 12:**Bibliometric study of fissure sealants between 1990-2022. Identification of current trends

**Adela Baca García, Pilar Valderrama Baca, Adriana Carolina Celi Maldonado**

 Introduction:

 The aim of this bibliometric research was to identify and analyse the 100 publications on fissure sealants (FS) with the highest Relative Citation Ratio (RCR) and the highest number of citations.

Material and Methods:

 A bibliometric study was conducted in PubMed, iCite, and Web of Science (WoS) Core Collection to identify and analyse the 100 articles with the highest RCR and citations respectively, published from 1990 to 2022. The data was downloaded on March 14, 2024.

Results:

 The 100 most influential articles according to RCR and citations were identified, and of these, 75 were common in both lists. The article with the highest RCR had a value of 25.98, and the one with the most citations had a value of 708 in iCite and 811 in WoS. Recent articles investigating trending topics in FS such as bioactive materials with antimicrobial properties were identified. In the list of the most influential according to RCR, the most represented journals were J Am Dent Assoc and J Dent with 10 documents each, most of the documents were oriented towards human biology and the prevailing design were in vitro studies.

Conclusions:

 The articles with the highest RCR are more recent than the articles with the highest number of citations. The combination of traditional metrics (citations) and others normalized in time and field, such as RCR, can offer a more robust view of research trends in the area of fissure sealants.

### **ORAL COMMUNICATION 13:**Oral health status in patients with cerebral palsy

**Paula Rilo Martínez, Eliane García Mato, Carolina Alejandra Muñoz Navarro, Clara Rodríguez Díaz, Iván Varela Aneiros, Jacobo Limeres Posse**

 Introduction:

 Patients with cerebral palsy have numerous physical (e.g. poor autoclisis), functional (e.g. dysphagia conditioning food texture) and/or sometimes behavioural (e.g. reluctance to routine oral hygiene) characteristics, which can potentially condition their oral health status.

Objectives:

 To assess the oral health status of a group of individuals with cerebral palsy attending a day centre where oral hygiene techniques are regularly applied.

Material and Methods:

 Oral examinations were performed on 77 individuals with cerebral palsy belonging to the Amencer-ASPACE Association (Vigo, Spain), evaluating the cod/CAOD index by age group. The results obtained were compared with the values of the general Spanish population collected from the “Oral Health Data Bank Insights” database (University of Valencia) in 2020.

Results:

 In children under 12 years old, the rate of active caries was higher in individuals with cerebral palsy compared to the general population (0.7 vs. 0.1). At 15 years old, the cod/CAOD rate was similar (1.0 vs 0.9). In the 35-44 age range, the prevalence of fillings in individuals with cerebral palsy was 1.3 and in the general population 4.6, while the prevalence of missing teeth was 4.8 and 1.9 respectively.

Conclusions:

 In individuals with cerebral palsy, it is necessary to promote oral hygiene habits from an earlier age and to provide more conservative dental procedures in adults.

### **ORAL COMMUNICATION 14:**Delphi study of oral healthcare for patients with special needs in Spain

**Antonio Expósito Delgado, Maria del Carmen Trullols Casas, Marta Lázaro Cabello, María Belén Ruiz Mancebo, Manuel Ribera Uribe**

 Introduction:

 Disabled or institutionalised people with co-morbidities and polymedication find it difficult to participate fully and effectively in oral services.

Objectives:

 To analyse the trend in oral health care for patients with special needs. To explore the actions needed to ensure safe and quality provision.

Material and Methods:

 A Delphi study was designed using a group of 20 experts. The criteria for inclusion in the selection were: representatives of scientific associations, representatives of Spanish Universities, the General Council of Dentists, Public Administration and external agents. A questionnaire was sent out on the current and future situation of care for patients with special needs. Consensus or agreement was established on responses obtaining a percentage higher than 80%, in 2 rounds of surveys.

Results:

 The prevalence of care needs and the demand of the special needs population will increase, although oral health will not improve. Promotion and prevention programmes could favour the stabilisation of oral health. It would be necessary to increase the benefits of the NHS service portfolio, although this would hinder its sustainability. Collaboration between universities and the NHS and mixed models of provision could favour efficiency. Techniques such as general anaesthesia are complex and costly, present risks and side effects, and should be indicated when alternatives such as behavioural control, stabilisation or outpatient conscious sedation fail, although this technique generates insecurity or discrepancies among experts. Training and research in gerontology, minimally invasive dentistry and oral medicine should be strengthened. The implementation of inclusive policies presents high-moderate difficulty.

### **ORAL COMMUNICATION 15:**Comparison of intra-examiner agreement in the diagnosis of caries lesions in vivo with and without loupes using the ICDAS II system

**Manuel De Feo Tolosa, María del Rosario Garcillán Izquierdo, Germán García Vicent, Ana Leticia Lenguas Silva, Jose Francisco Martín Morales, María Victoria Mateos Moreno**

 Introduction:

 The current management of caries considers new non-operative strategies where diagnosis plays a leading role. Dental loupes are a tool to consider for the diagnosis of carious lesions; however, there is no conclusive literature.

Objectives:

 This cross-sectional in vivo study compares the agreement, sensitivity, and specificity of the diagnosis of carious lesions with and without loupes.

Material and Methods:

 A single examiner inspected 143 occlusal surfaces. For each surface, two measurements of carious lesions were taken with 3.5x magnification loupes and two measurements without loupes. There was a gap of 7 to 30 days between each measurement. The ICDAS II diagnostic method was used for occlusal surfaces to determine the stage of carious lesions. A sample size calculation was performed with an alpha error of 0.05 and a statistical power of 80%, taking an expected effect size of 0.2. Statistically, a weighted intraexaminer Cohen's Kappa index was calculated, along with sensitivity and specificity measurements for types of incipient, moderate, and severe lesions.

Results:

 The intra-examiner agreement with the use of dental loupes was 0.88, while the agreement without loupes was 0.81. For the diagnosis of incipient lesions, a sensitivity of 0.92 and a specificity of 0.6 were achieved.

Conclusions:

 The precision in the diagnosis of caries using loupes is similar to the precision without them. In the case of incipient lesions, a high sensitivity is achieved with the use of loupes.

### **ORAL COMMUNICATION 16:**Analysis of the care capacity of the dental service at the central prison in Yaoundé, Cameroon

**David González Alarcón, Bell Isabelle Pulcherie Ngo Masso, Michael Agbor Ashu, Marcelle Aurore Mankongo, Julia Sánchez Ituarte, Bachelard Juvan Dongmo**

 Introduction:

 The Cameroonian public health system does not provide dental care for the inmate population. Oral pathologies have a high impact on the total burden of disease and on the quality of life.

Objectives:

 To assess the dental care capacity in the central prison of Yaoundé.

Material and Methods:

 Quantitative and qualitative cross-sectional study. Duration: 18 months. Open interview and calibrated survey of inmates who were treated by the medical team of the association “Circular Research”.

Results:

 256 (5.3% of inmates) were consulted and 245 (5.1%) of them received at least one oral treatment. Among the treatments performed, 305 (88.4%) were extractions, 29 (8.4%) application of cariostatics, 10 (2.9%) atraumatic restorative treatments, and 1 (0.3%) basic periodontal treatment. 90.5% of participants considered that the treatments they received had significantly improved their quality of life, and 97.6% believed that dental treatments should be a priority in the prison health system.

Conclusions:

 The high burden of oral disease should be considered as an important axis of care for the prison population. Currently, the Cameroonian prison system does not consider access to oral health as a priority, with social initiatives responding to this need in a residual way. It is necessary to work on a specific agenda on oral health and the prison population in Cameroon in order to develop formulas to implement a comprehensive action plan.

### **ORAL COMMUNICATION 17:**The activity of specialised paediatric dentistry services in primary care in Vallès Oriental (Barcelona)

**Elias Casals Peidró**

 Introduction:

 The conservative dentistry activity is covered by the National Health Service in Catalonia and provided in primary care centres. In the Granollers- Mollet Primary Care Service, two specific units were created to carry out this activity which are located in Granollers and Mollet being named Children's Specialized Dentistry Services (SOEI).

Objectives:

 To evaluate the conservative dentistry activity carried out in the last 20 months (January 2023-September 2024) in the specialized paediatric dentistry services of the Granollers-Mollet territory (province of Barcelona). Assess patient engagement by exploiting data from scheduled visits in which the patient has not shown up or cancelled their visit.

Material and Methods:

 The activity noted (fillings and seals) has been exploited by the professionals who practise in the SOEI, who use the eCAP-HES programme to reflect the activity carried out. The delay in SOEI appointments throughout the weeks of the study has been evaluated.

Results:

 The results show the progressive increase in conservative activity due to the increase in the number of days of the SOEI staff Works as well as the increase in the number of patients visited per day from 8 to 9. The average monthly amount of fillings placed at the dental service in Granollers was 48,25 in 2023 and 77 in 2024.

Conclusions:

 The capacity to carry out conservative activity has progressively increased.

### **ORAL COMMUNICATION 18:**Is prenatal exposure to endocrine disrupting chemicals (phthalates and bisphenols) associated with Molar Incisor Hypomineralization? A prospective cohort study

**Ester Cots Corominas, Francisco Antonio Guinot Jimeno, Pere Toran, Lea Kragt, Nuria Güil-Oumrait, Maribel Casas, Martine Vrijheid**

 Introduction:

 Molar incisor hypomineralization (MIH) is a defect in the mineralization of the first permanent molars and incisors. The prevalence of MIH in children is approximately 14%, and its etiology is still uncertain. Bisphenol A and di(2-ethylhexyl) phthalate (DEHP) exposure have been associated with enamel defects in animal studies, but these associations have not been studied in humans.

Objectives:

 To assess the association between urinary bisphenol and phthalate concentrations during preg nancy and presence of MIH in adolescents.

Material and Methods:

 A total of 228 mother-child pairs from INMA Sabadell cohort study were included. Concentrations of phthalates and bisphenols were determined in first and third trimesters urine samples. Presence of MIH was assessed through a parental reported questionnaire at 15 years of the adolescent. Multivariate logistic regression models adjusted for covariates were used.

Results:

 The prevalence of MIH was 5.3%. Exposure to mono(2-ethylhexyl) phthalate (MECPP) concentrations during the third trimester of pregnancy were associated with a reduced risk of MIH in the overall population (adjusted odds ratio (ORadj) for an interquartile-range increase in log2-transformed concentrations=0.54, 95% CI=0.31-0.91). In boys, exposure to DEHP metabolites in the first trimester, mono-2-ethyl-5-hydroxyhexyl phthalate (mEHHP) and mono-2-ethyl-5-oxohexyl phthalate (mEOHP) tended to increase the risk of MIH (mEHHP: ORadj=1.73, 95%CI=0.97-3.08).

Conclusions:

 This study suggests that prenatal exposure to phthalates may be associated with MIH. Further studies on environmental chemical exposures may help to identify the mechanisms behind MIH.

### **ORAL COMMUNICATION 19:**Social prescription in primary care dental consultation: regarding two clinical cases

**Agnès Masgrau, Marta Amela Morcillo, Marta Pulido Martí, Judit Madevall Galter, Judit Joseph Fortuny**

 Introduction:

 Social prescribing is a mechanism through which a health professional and a patient identify together community activities to improve their health and well-being. Classically, prescribers are doctors, nurses or social workers. This communication aims to highlight the dentist as another member of the primary care team and therefore, a potential social prescriber from the dental consultation.

Description of the clinical cases:

 We present two clinical cases with a common link: two adult women, who come to the primary dental clinic with reasons for consultation for dental pain, apparently with different diagnoses, although both cases turned out to be myofascial pain due to temporomandibular dysfunction, with a clear emotional and psychological component. We present how, in addition to providing pharmacological treatment, hygienic measures and adjuvants, we can prescribe a community resource or a group health education activity, which goes beyond mere oral health and encourages healthy lifestyle habits for prevention and promotion of their health.

Conclusions:

 The importance of clinical diagnosis taking into account the social determinants of health. Treatment centred on the person, contextualized, with a salutogenic and equitable vision. Empower people to manage their own health. Take advantage of the resources and assets that the community offers. Participate as dentists in the comprehensive and integrated treatment of the population from primary care consultations.

### **ORAL COMMUNICATION 20:**Characterisation of the periodontal proteome in gingival crevicular fluid and saliva in periodontitis by SWATH-MS

**Triana Blanco Pintos, Iryna Kuz, Noelia Seijas Otero, Alba Sánchez Barco, Carlos Balsa Castro, Inmaculada Tomás**

 Introduction:

 Few studies have evaluated the structure of the periodontal proteome and the protein expression in gingival crevicular fluid (GCF) and saliva.

Objectives:

 To analyse the proteome structure and to compare the expression of proteins quantified in GCF and saliva in periodontally healthy subjects and with periodontitis.

Material and Methods:

 GCF and saliva samples were collected from 44 healthy periodontal subjects and 41 with periodontitis (stages III-IV)1. The samples were analysed by sequential window acquisition of all theoretical mass spectra (SWATH-MS) and the proteins were identified using the human UniProt database2. The periodontal proteome structure was assessed using principal component analysis (PCA). Protein expression was considered different for an adjusted p-value <0.01 and an absolute effect size >0.2

Results:

 A total of 250 proteins in GCF and 377 in saliva were quantified. The proteome structure was different in periodontitis compared to periodontal health in both sample types. Considering the protein expression in GCF, 115 (46.0%) were differentially expressed, with 61 (53.0%) being upregulated and 54 (47.0%) downregulated in periodontitis. In saliva, these numbers were: 108 (28.7%), 82 (75.9%) upregulated and 26 (24.1%) downregulated.

Conclusions:

 Periodontitis modifies the periodontal proteome structure and the expression of numerous proteins in GCF and saliva. The differential expression patterns are different in both media, with a balance of upregulated and downregulated proteins in GCF, while in saliva, overexpressed proteins are predominant.

### **POSTER 1: **Use of public dental services among the adult population in Galicia

**Carmen García Seijas, Marta Mulero de Caso, Paula Fernández Riveiro, Almudena Rodríguez Fernández**

 Introduction:

 Oral diseases are a public health problem due to their prevalence and the cost associated with their treatment. The integration of this into primary care services is an important issue for all countries.

Objectives:

 To analyse the use made by the Galician adult population of public oral health care services, as well as to ascertain other characteristics of their use (degree of knowledge of the services, reasons for nonuse, satisfaction...).

Material and Methods:

 A cross-sectional study was carried out through an online questionnaire, consisting of 11 questions, addressed to the Galician population of legal age. The link was distributed through the Whats- App messaging service.

Results:

 52 people took part in the study and the average age was 38 years old. Fifty percent of the individuals were aware of the existence of the service and of these, 62% were aware of the benefits. Among those aware of the units, 46% had not considered using them and the main reason for this was satisfaction with their usual private clinic (71%). The most frequently performed procedures were revision and sealing (54%). All users rated their experience in these units as good or excellent.

Conclusions:

 These results suggest that the use of public dental services by the Galician adult population is low and that they are mainly used during adolescence. It would therefore be necessary to improve knowledge through information campaigns.

### **POSTER 2: **Analysis of plasma cell-free DNA in head and neck cancer diagnosis using different quantification approaches

**Ana María Rodríguez Ces, Santiago Agüín, Inés Formoso García, Rafael López López, María Mercedes Suárez Cunqueiro**

 Introduction:

 in recent years, circulating cell-free DNA (cfDNA) has emerged as a promising biomarker for various types of cancer, including head and neck cancer (HNC). This study aims to evaluate and compare two methods for cfDNA quantification—fluorometry and polymerase chain reaction (PCR)—in patients with HNC and healthy individuals, to assess their diagnostic utility.

Material and Methods:

 plasma samples from 30 patients with locally advanced HNC and 15 healthy controls were analysed using both techniques. The results showed a significant correlation between the two quantification methods.

Results:

 fluorometry proved highly effective in distinguishing between patients and healthy controls due to its ability to detect higher concentrations of cfDNA. On the other hand, PCR demonstrated greater precision in detecting low levels of cfDNA, which could be useful for identifying early-stage cases or those with a low tumor burden.

Conclusions:

 these findings suggest that combining both methods could enhance the accuracy of HNC diagnosis and monitoring, facilitating early detection and disease tracking.

### **POSTER 3: **Use of artificial intelligence for decisionmaking in orthodontic extractions. Systematic review

**Ulises Armando Villasanti Torales, Julieta Maria Mendez Romero, Julio Cesar Mello Roman, Ana Liesel Guggiari Niederberger, Marta Ines Ferreira Gaona, Jose Luis Vazquez Noguera**

 Introduction:

 One of the most debated decisions in orthodontics is the need for premolar extractions. Artificial Intelligence (AI) can be of great help in improving orthodontic treatment planning.

Objectives:

 Study aims to evaluate the accuracy of AI methods to predict the need for tooth extractions in orthodontic treatment.

Material and Methods:

 We conducted an electronic search in the databases: MEDLINE (PubMed), Embase, Web of Science, Scopus, Google Scholar (for grey literature), LILACS, ACM Digital Library published until April 2024. A systematic synthesis of data was performed.

Results:

 The AI methods used were Neural Network and Random Forest in most cases. The most predictive characteristics were the crowding or availability of space in the upper jaw, the position of the upper and lower incisors in relation to their bone bases, the proportion of posterior and anterior facial height, and the anterior facial height itself. Overall, all included studies were 78.6% accurate. The decision tree had an accuracy of 86.08%, followed by the neural network 83.45% and the random forest 83.04%. Regarding the type of input, the use of clinical and cephalometric data had an overall accuracy of 93.34%, followed using occlusal photographs with an accuracy of 91.39%.

Conclusions:

 AI has the potential to significantly improve the accuracy and efficiency of orthodontic removal decision-making, but its implementation must be carefully managed and always complemented by professional judgment to ensure the best outcomes for patients.

### **POSTER 4: **Relationship between salivary lactoferrin and tooth decay in individuals with Down syndrome

**Aldara Alonso Barciela, Eliane García Mato, Desirée Antequera Tienda, Eva Carro Díaz, Lucía Sande López, Iván Varela Aneiros**

 Introduction:

 Scientific evidence supports that people with Down syndrome exhibit a lower prevalence of dental caries than the general population. It has been suggested that reduced levels of salivary lactoferrin, a glycoprotein with antibacterial activity, may contribute to the development of caries in non-syndromic individuals.

Objectives:

 To assess the salivary lactoferrin concentration in individuals with Down syndrome, with and without caries.

Material and Methods:

 A convenience study group of 72 individuals with Down syndrome was established, of whom 9 had active caries (ICDAS >2) and 63 did not have caries. Unstimulated saliva samples were collected from all participants and processed to determine lactoferrin concentration using a specific ELISA kit (ab108882, Abcam, Cambridge, UK), total protein concentration (bicinchoninic acid [BCA] assay) using the BCA protein assay kit (Pierce, Rockford, IL, USA) and the lactoferrin/BCA ratio. Results were analysed by Wilcoxon-Mann-Whitney U test.

Results:

 Lactoferrin levels were significantly elevated in individuals with caries (median= 12.04 μg/mL (Q1= 8.57; Q3= 12.76) compared to those without caries (median= 7.04 μg/mL [Q1= 4.41; Q3= 10.39]) ( p= 0.038). No differences in BCA or ratio lactoferrin/BCA levels were detected between individuals with and without caries.

Conclusions:

 Lactoferrin might play a relevant role in caries susceptibility in Down syndrome.

### **POSTER 5: **Habits vs. Knowledge about Tooth Brushing in Children and Adolescents from Orense

**Lorena Quintas Fírvida, Yolanda Fernández González, Cristina Rodríguez Escuredo, María Jesús Pereira Gil, María Asunción Pérez López, Miguel Franco Pérez**

 Introduction:

 Oral health habits acquired during childhood and adolescence are crucial for the prevention of oral diseases throughout life. Tooth brushing is a fundamental practice in this context.

Objectives:

 To evaluate and contrast the level of knowledge and oral hygiene habits in young people from Orense, analysing differences according to sex and educational level.

Material and Methods:

 A cross-sectional study was conducted within the framework of the “Look at that mouth” project during the 2023/2024 academic year. 2000 students (1327 from 4th grade of Primary and 673 from 2nd year of ESO: Secondary Education) from 63 educational centres in Orense participated. A questionnaire on oral hygiene knowledge and habits was applied. A descriptive and comparative analysis was performed.

Results:

 A significant discrepancy was observed between oral hygiene knowledge and practices. Adolescents showed a tendency to overestimate their knowledge and habits. Statistically significant differences were identified according to sex and educational level.

Conclusions:

 There is an “infantilization of oral hygiene”, with a gap between theoretical knowledge and actual practices, especially in adolescents. It is necessary to reinforce oral health education at all educational levels, with emphasis on the adolescent population.

### **POSTER 6: **Randomized crossover clinical trial on the substantivity of a single application of chlorhexidine and o-cymen-5-ol gel on oral biofilm and saliva

**Noelia Seijas Otero, Berta Suárez Rodríguez, Iryna Kuz, Triana Blanco Pintos, Alba Regueira Iglesias**

 Introduction:

 No clinical trials have evaluated the antimicrobial activity and substantivity of gel formulations containing chlorhexidine (CHX) and cymenol1.

Objectives:

 To compare the in situ antimicrobial effect and substantivity of a 0.20% CHX + cymenol gel (test) with 0.20% CHX gel formulation (control) on salivary flora and dental plaque biofilm up to seven hours after a single application.

Material and Methods:

 Twenty-nine orally healthy volunteers were selected for the development of Experiments 1 (saliva) and 2 (dental plaque biofilm). All subjects were randomly assigned to receive either the test or control gels. Samples were collected at baseline and five minutes, one, three, five, and seven hours after a single application of the products. The specimens were processed using confocal laser scanning microscopy. Bacterial viability (BV) was quantified in the saliva and biofilm samples. The BV was calculated using the DenTiUS Biofilm software.

Results:

 The mean baseline BV was significantly reduced five minutes in both Experiments after application of the gel test (E1: 87.00% vs 26.50%; E2 total thickness of biofilm: 91.00% vs 5.80%; E2 upper layer: 91.29% vs 3.94%; and E2 lower layer: 86.29% vs 3.83%; p<0.01 for all experiments). This effect was maintained after seven hours (40.40%; 21.30%; 24.13%; and 22.06%, respectively; p<0.01 for all experiments). The control group showed similar results in both experiments and sample times (p<0.01 in all comparisons).

Conclusions:

 A single 0.20% CHX and cymenol gel application exerts potent and immediate antimicrobial activity on saliva and dental plaque. This effect is maintained seven hours after application.

### **POSTER 7: **The postcode as a determinant of oral health

**Josep Busquet Vila, Ana Jesús Herrero Carrión, Marta Figueras Cabanes, Ruth Martí Lluch, Eric Tornabell Noguera**

 Introduction:

 The primary health care area Girona 3 provides primary health care services on both sides of the Onyar River. The care is provided in two health centres, one on each side of the river. Despite being assigned a common morbidity situation and socioeconomic indicator, the characteristics of the population and treatment needs vary according to the postal code.

Objectives:

 To determine if there are differences in oral health indicators among the school-aged children in the schools on each side of the river.

Material and Methods:

 Analysis of oral health variables from mixed, public, and semi-public educational centres, in the cohorts reviewed in Catalonia. Variables: Dependent: Percentage of the population without caries experience in the grades: preschool 4, 1st, and 6th grade. dmft in preschool 4 and 1st grade. DMFT in 6th grade. Independent: Location of the educational centre (zone A and B).Fisher’s test is used for comparing the percentage of healthy population, and the Wilcoxon test is used to compare the means of DMFT and dmft.

Results:

 The percentage of the child population without caries experience: 76.56% for zone A and 20.76% for zone B. The dmft in preschool 4 is 0.63 in zone A and 5.02 in zone B.

Conclusions:

 The oral health of the school-aged population in zone B is different from that in zone A. It is necessary to implement individualized oral health promotion and prevention measures by zones.

### **POSTER 8: **Systematic review of the use of aligners in growing patients

**Ana Leticia Lenguas Silva, Mª Victoria Mateos Moreno, José Francisco Martín Morales, Germán García Vicent, Manuel De Feo Tolosa, Mª Rosario Garcillán Izquierdo**

 Introduction:

 Clear aligners, introduced in 1999, have become popular among adults and recently in children and adolescents. Mixed dentition is a critical phase due to its orthopaedic potential, arch development, and malocclusion interception. Early treatment can reduce the duration and complexity of future orthodontic treatments.

Objectives:

 To review and analyse available literature on indications, efficacy, advantages, and disadvantages of aligner use in growing patients.

Material and Methods:

 A search was conducted in PubMed and Cochrane Library using the following keywords “aligners”, “orthodontic”, “growing patients”, “mixed dentition”, “interceptive orthodontics”, “children”, “early treatment”, “class II malocclusion”, “mandibular advancement”. Twenty-nine articles published between January 2014 and September 2024 were included.

Results:

 Studies on aligners in growing patients are scarce, mainly observational and retrospective. Limited evidence was found on their efficacy in correcting malocclusions and their impact on periodontal health compared to traditional therapies.

Conclusions:

 More controlled clinical trials with larger samples are needed to improve scientific evidence on early intervention with aligners. Clarification is necessary regarding their indications as interceptive orthodontics, efficacy in malocclusion correction, relapse prevalence, and impact on oral health.

### **POSTER 9: **Do social networks influence patients undergoing cosmetic dental treatment?

**Julia Sánchez Ituarte, Yolanda Freire Mancebo, Margarita Gómez Sánchez, Manuel Frías Senande, Víctor Díaz-Flores García, Ana Suárez García**

 Introduction:

 In recent years, there has been a significant increase in the use of social media. It is important to explore the impact that these may have on patients undergoing aesthetic dental treatments.

Objectives:

 to investigate the influence of social media on patients when undergoing aesthetic dental treatments.

Material and Methods:

 cross-sectional study using an online survey. Participants were students over 18 years who were users of social media and had undergone aesthetic dental treatment. Data were evaluated using Pearson’s chi-square test. The level of significance was set at p <0.05.

Results:

 504 responses were obtained, of which 148 (29.37%) indicated that they had been influenced by social media. No significant difference was found in the influence based on the social media or the number of those used. No differences were observed based on the type of treatment. Significantly, these participants used social media more frequently at night. 70.9% of these participants indicated that they had searched for information about the aesthetic treatment they had undergone on social media.

Conclusions:

 Social media can influence some patients’ decision to undergo aesthetic dental treatment. Dental professionals should consider the growing importance of this type of publication in their daily practice.

### **POSTER 10: **Reading nutritional labels in the supermarket: A group health education activity

**Sara Fradera Barbe, Cristina Mas Serra, David Suros Torras, Judit Masdevall Galter, Lidia Roca Mussoll**

 Introduction:

 The consumption of soft drinks is one of the main sources of added sugars in the diets of children and adults. According to a 2023 study, Spanish children aged 7 to 12 consume an average of 55.7 g/ day of sugar, with cookies, cocoa powder, and sugary yogurts being the most commonly consumed products. To address this issue, a group intervention has been designed to provide patients with the skills to interpret nutritional labels, as cognitive-behavioural interventions are effective for changing habits.

Objectives:

 The objectives are to explain labelling according to regulation 1169/2011, differentiate types of sugar, raise awareness of the problems associated with sugar consumption, and promote healthier purchasing decisions.

Material and Methods:

 The approach includes theoretical concepts and practical label-reading exercises in large stores and small businesses, targeting patients with excess weight (BMI > 24.99) who are motivated to improve their habits, while excluding those with language barriers or mental Health disease.

Results:

 The PREDIMED questionnaire is used to assess dietary habits and participant satisfaction. 

Results:

show an increase in fruit and vegetable consumption and a decrease in ultra-processed foods following the intervention.

Conclusions:

 The group activity provides tools for better product selection in supermarkets and helps identify hidden sugars, contributing to a reduction in daily sugar consumption and alignment with WHO recommendations.

### **POSTER 11: **Melatonin gummies as a risk factor for the development of dental caries

**Carlos Alberto Vivas Mefle, Ángel Orión Salgado Peralvo, María Rosario Garcillán Izquierdo, Bibiana Mateos Moreno, Lorenzo Dorado Jara, María Victoria Mateos Moreno**

 Introduction:

 In recent years, the consumption of melatonin to induce sleep has increased, becoming the most frequently consumed substance by the child and adult population.

Objectives:

 To investigate the composition of “melatonin gummies” (MG) and whether there are sugar-free alternatives with anticariogenic substances.

Material and Methods:

 A search for “melatonin gummies” was performed on Google.com

Results:

 Fifty-one different MG brands were found, of which 35 contained sugar (68.6%). Depending on the brand, the sugar content per gummy varies between 1.000-4.000 mg, while the melatonin content varies between 0.5-10 mg. 29.4% (fifteen products) do not specify the amount of sugar in their composition, and 4 are called by the manufacturers as “no added sugars”, despite containing maltodextrin which reduces the pH of the oral biofilm and have the potential to demineralise tooth enamel. The most frequently observed sugars and in the highest proportion are sugar and glucose syrup and, to a lesser extent, cane sugar, tapioca syrup, maltodextrin, corn syrup, sucrose, dextrose, beet sugar and tapioca sugar. These sugars are found in the ingredients of practically all MG in the first and second positions, so they are their majority components.

Conclusions:

 A high proportion of MG products available on the market contain high concentrations of sugar, so the general population should be advised not only to consume sugar-free MG products, but also those containing anticariogenic substances.

### **POSTER 12: **Incorporation of the 0-5 age group into the primary care dentistry portfolio in Catalonia

**Cristina Vida Lucea, Marta Figueras Cabanes, Elisabet Caula Pinsach, Manuel Quintana Montero, Eva Mora Beneyto**

 Introduction:

 The inclusion of the 0 to 5-year-old population group in the common portfolio of primary care dental services has necessitated the review and update of the paediatric dental care protocol in Catalonia. This update includes preventive visits for certain cohorts, criteria for identifying children at high risk of caries, and preventive activities for these patients. Health and monitoring indicators have been developed for the incorporated activities, some of which have been adopted as quality standards.

Objectives:

 To determine the prevalence of caries in various cohorts. To analyse the impact of standardising preventive activities.

Material and Methods:

 A descriptive analysis of the data from the information system has been carried out from June 2023 to August 2024. Oral health indicators: • Prevalence of caries in the 0 to 2-year-old population. • Prevalence of caries in the 5-year-old population. Care quality standards: • Percentage of the population under 24 months reviewed by a dentist. • Percentage of the 5-year-old population reviewed by a dentist. • Percentage of the 0 to 5-year-old population at risk of caries who received preventive follow-up.

Results:

• Caries prevalence in the 5-year-old population: 27.03%. • Review coverage at 5 years: 81.01%. • Preventive follow-up among 0 to 5-year-olds: 44.70%.

Conclusions:

 The implementation of school-based reviews for the 5-year-old cohort has achieved coverage of more than 80% in this group. The number of highrisk children aged 0 to 5 receiving preventive follow-up has doubled.

### **POSTER 13: **Monitoring the incorporation of dental hygienists into primary care teams in Catalonia (2022- 2024)

**Elisabet Caula Pinsach, Marta Figueras Cabanes, Cristina Vida Lucea, Manuel Quintana Montero, Eva Mora Beneyto**

 Introduction:

 The monitoring of preventive activity in primary dental care teams in Catalonia in 2021 revealed a low capacity to carry out the expected activities. This argument led to the incorporation of dental hygienists into primary care teams.

Objectives:

 To evaluate the incorporation of dental hygienists into the service portfolio. To evaluate the incorporation of dental hygienists in the quality standards for primary dental care teams in relation to the child population at risk of caries.

Material and Methods:

 Observational study of data recorded in primary care information system from 2021 to present. Indicators described: Number of patients with activities carried out (sealing and fluoridation). Percentage of participation of hygienists in these activities. Quality standards: percentage of child population at risk of caries and presence of sealings. Percentage of population with early childhood caries with preventive follow-up.

Results:

 The number of patients receiving annual sealings has increased by a factor of 15; 84% are now performed by hygienists. The number of patients receiving annual fluoridations has increased by a factor of 4; 64.21% are now performed by hygienists. Preventive follow-up of patients with early childhood caries has increased by more than 21 percentage points, with hygienists involved in 52% of cases.

Conclusions:

 The increase in the participation of hygienists in preventive activities has consolidated them as an essential professional in the team. The evolution of the activities shows the turning point that coincides with their incorporation.

### **POSTER 14: **Healthy habits and sustainability in students of dentistry and human nutrition and dietetics

**Teresa Almerich Torres, José Manuel Almerich Silla, Ana Ruiz Miravet, José Enrique Iranzo Cortés, José María Montiel Company, José Miguel Soriano del Castillo**

 Introduction:

 Diet has been identified as a determining factor in the development of both oral and systemic non-communicable diseases (NCDs). In 2015, the United Nations approved 17 Sustainable Development Goals (SDGs). SDG 3 aims to promote healthy lives and well-being at all ages. SDG 12 aims to change the current model of production and consumption to achieve efficient resource management and reduce waste generation. As part of an educational innovation project, an intervention was carried out on Dentistry and Human Nutrition and Dietetics students at the University of Valencia using ICTs to expand their digital competencies.

Objectives:

 The objective was to raise awareness and expand students’ knowledge about the implications of their dietary habits and consumption practices to improve their health and become more sustainable.

Material and Methods:

 To measure the degree of knowledge acquired through this methodology, students completed a questionnaire before (n=91) and after participating in the activities (n=79).

Results:

 No statistically significant differences were observed in the final grades of students from both degree programs, but differences were observed (p>0.001) between the grades obtained in the questionnaires conducted before carrying out the project activities and those after, in both degree programs.

Conclusions:

 Students have increased their knowledge in the subject, as revealed by the survey results. We propose the formulation of more teaching interventions that go beyond the framework of traditional lectures aimed at promoting compliance with the SDGs in higher education.

### **POSTER 15: **Burnout assessment in dentistry students at the University of Valencia

**José María Montiel Company, José Enrique Iranzo Cortés, Teresa Almerich Torres, José Manuel Almerich Silla, Inmaculada Perelló Berenguer**

 Introduction:

 Dental students are subjected to a series of conditions that can cause them to develop stress and fatigue, making them prone to developing burnout syndrome (BOS). The increasing number of cases has sparked interest in the academic and health fields.

Objectives:

 To assess the presence of BOS at two points in the dental degree (2nd and 5th year) and to evaluate how gender affects it.

Material and Methods:

 149 students (79 from the 2nd year and 70 from the 5th year) completed the Maslach Burnout Inventory- HSS through the LimeSurvey platform. Means were calculated for each domain and group, and comparisons were made using the t-student test. Prevalence was also assessed, and differences between years and gender were analysed using the Chisquare statistic.

Results:

 Mean score for the emotional exhaustion (EE) domain was 25.70 points in the 2nd year; 8.32 for depersonalization (D), and 26.80 for personal accomplishment (PA). In the 5th year, the values were 28.97, 8.36, and 33.40, respectively. Prevalence of EE was found in 50.6% of 2nd-year students and 67.1% of 5th-year. D values were 45.6% and 44.3%, and PA values were 75.9% and 60.0%, in the 2nd and 5th years, respectively.

Conclusions:

 Prevalence of burnout was high. A significant difference was found between second and fifth-year students in terms of EE, with higher levels in fifth-year, and PA, with lower levels in fifth-year. The gender study found significant differences in EE and D in the fifth-year, with higher levels in women in both cases.

### **POSTER 16: **Monitoring the implementation of silver diammine fluoride treatment in primary care dental surgeries in Catalonia

**Marta Figueras Cabanes, Elisabet Caula Pinsach, Eva María Mora Beneyto, Manuel Quintana Montero, Cristina Vida Lucea**

 Introduction:

 In 2023, the Department of Health protocolized and planned activities to incorporate silver diamine fluoride (SDF) treatment into primary care dental consultations. These activities included informational and follow-up meetings with the directors and managers of health regions, training for professionals, and the publication and dissemination of the implementation protocol. Collaboration was established with the technical team of the Primary Care Services Information System to design the necessary indicators for monitoring. Objective: To analyse the implementation of SDF treatments in the dental consultations of Primary Care Teams in Catalonia.

Material and Methods:

 Review of the minutes from meetings with health regions regarding the interventions related to the deployment of SDF treatment. Analysis of activity by health region and primary care teams: - Number of teams where the activity has begun. - Number of patients receiving SDF treatment for primary and permanent teeth - Health Impact Analysis: • Incidence of dental abscess diagnosis • Annual number of extractions of primary teeth

Results:

 The factors influencing the implementation of the treatment have been logistical and human. Activity indicators: The number of patients receiving treatment has clearly increased each month and varies by health region. Activity has begun in 81 out of 378 teams.

Conclusions:

 Implementation has taken place across all ten health regions, but not equitably. Currently, the use of this therapeutic tool is dependent on professional discretion. It is still not possible to assess the health impact of the treatment.

